# Unusual clinical presentation of cutaneous leishmaniasis in a patient with corticosteroid‐induced immunosuppression

**DOI:** 10.1002/ccr3.3482

**Published:** 2020-11-24

**Authors:** Maryam Mahdavi, Reihaneh Aryan, Yalda Nahidi, Mehrdad Teimoorian, Vahid Mashayekhi

**Affiliations:** ^1^ Cutaneous Leishmaniasis Research Center Mashhad University of Medical Sciences Mashhad Iran; ^2^ Golestan University of Medical Sciences Gorgan Iran

**Keywords:** cutaneous leishmaniasis, disseminated, immunosuppression, recurrence, systemic corticosteroids

## Abstract

Clinicians should always consider rare, atypical, and opportunistic infections in patients undergoing long‐term systemic corticosteroid therapy. Diagnosis needs further evaluations and special consideration.

## Graphical abstract

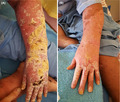

## 1 INTRODUCTION

Cutaneous leishmaniasis (CL) is often a localized and self‐limited disease, but its behavior changes in the state of immunosuppression. Here, we report a rare clinical presentation of disseminated CL after reactivation of leishmania infection in a 42‐year‐old man with corticosteroid‐induced immune suppression.

Cutaneous leishmaniasis is a parasitic infectious disease which is endemic in several countries around the world including Iran. This infection is caused by a group of protozoan parasites of the genus *Leishmania* and is transmitted by the bite of infected female phlebotomine sandflies.^1^

In Iran, CL is mostly caused by *Leishmania. major* and *Leishmania. tropica* species.^2^ The clinical features of CL depend on various factors including *Leishmania* species, environmental factors, genetic factors, and immune response of the host.^2^

Innate and adaptive immune responses play an important part in controlling leishmania infection, and immunosuppression is a known risk factor for unusual clinical variants of CL.^3^, ^4^

Corticosteroids are beneficial in treatment of a wide spectrum of diseases but their use has been shown to come with a number of well‐documented risks including development of serious bacterial infections, opportunistic infections, and also altering clinical manifestations of infectious diseases. Therefore, the risk‐benefit assessment must be borne in mind when long‐term corticosteroid therapy is considered.^5^

This case report highlights the unconventional clinical manifestation and reactivation of CL in a patient undergoing systemic steroid therapy and demonstrates that the diagnosis could be challenging as it mimics many other diseases.

## 2 CASE PRESENTATION

### 2.1 Case history/examination

In January 2019, a 42‐year‐old man presented with fever, extensive widespread skin lesions, and painful oral ulcers to the emergency department of Imam Reza hospital of Mashhad city, Iran.

**FIGURE 1:**
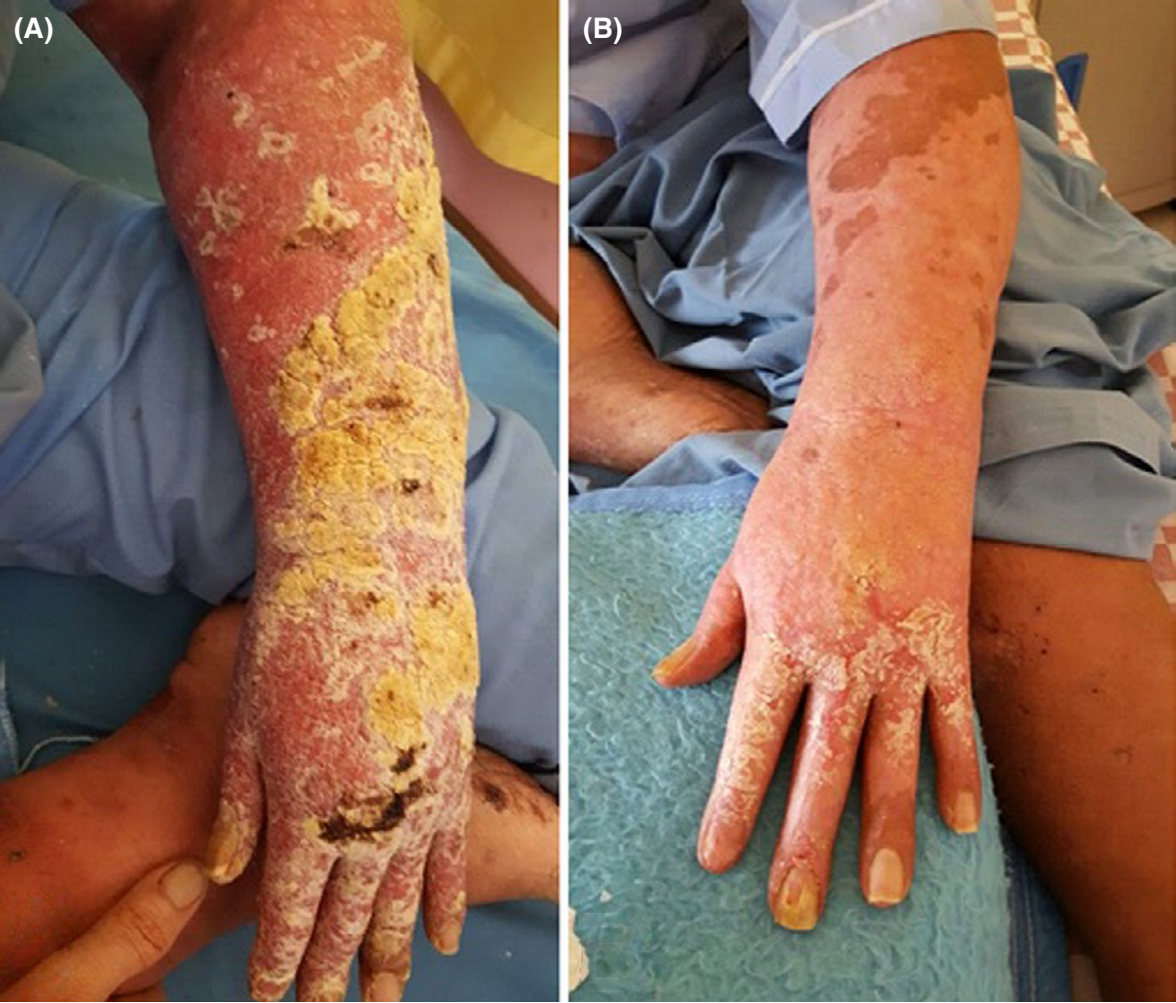
Erythematous and indurated lesions with thick hyperkeratotic crusts before treatment (A) and after 28 d of treatment with glucantime (B)

**FIGURE 2:**
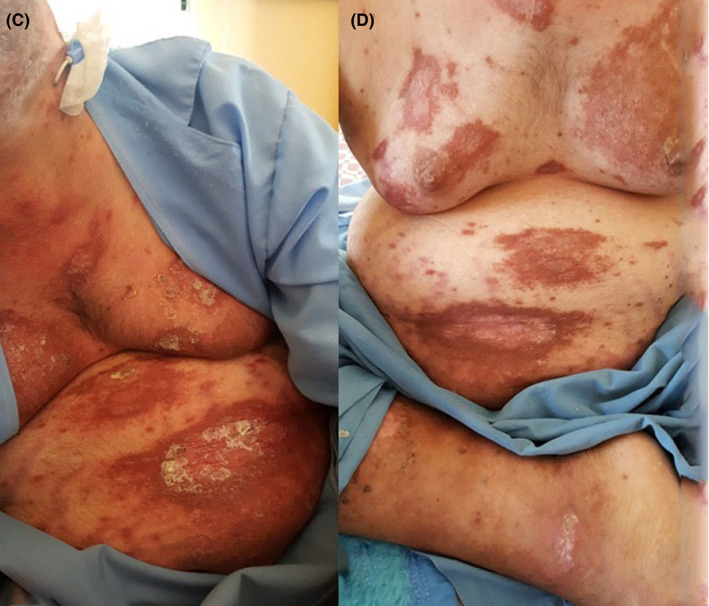
Cutaneous lesions on patient's torso before treatment (C) and after 28 d of treatment with glucantime (D)

In physical examination, he had a fever of 39°C, multiple ulcers on the soft palette and buccal mucosa, extensive reddish purple papules and plaques on the face, scalp, torso and limbs. The plaques in some areas were inflammated and ulcerated with serous discharge and some were erythematous with thick hyperkeratotic crusts (Figures 1 and 2). Also bilateral orbital edema, cushingoid appearance, and two atrophic scars on the right cheek were witnessed.

The lesions had started 2 years ago and ever since there were periods of remissions and recurrences. During the last few days, the patient had experienced deterioration of skin lesions and development of oral ulcers and fever.

There was a history of CL lesions on the face 4 years ago, and he had undergone treatment with intralesional antimoniate which was followed by partial remission.

The patient reported arbitrary use of prednisolone 30 mg per day due to rheumatoid arthritis (RA) for 10 years.

The patient was febrile and had some punched‐out ulcers on his soft palate causing dysphagia alongside extensive and widespread skin lesions which in some areas were ulcerated with serous discharge promoting a primary diagnosis of bacteremia status and reactivated HSV due to immunosuppression. Patient received antibiotics, oral acyclovir, and chlorhexidine mouthwash in the emergency department.

### 2.2 Differential diagnosis, investigations, and treatment

**FIGURE 3:**
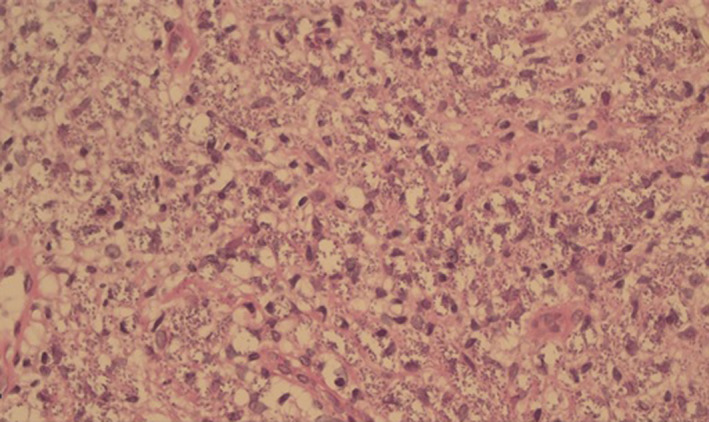
Hematoxylin and eosin stain showing dense lymphohistiocytic infiltrates in dermal layer and histiocytes with foamy cytoplasm and numerous leishman bodies

Biopsy was taken from skin lesions with variable differential diagnoses such as psoriasis, sarcoidosis, sweet syndrome and mycobacterial infections and connective tissue diseases. Histopathology showed dense lymphohistiocytic infiltrates in the dermis. The histiocytes had foamy cytoplasm with numerous leishman bodies inside (Figure 3).

Skin smears from four other lesions from different areas of the body were collected and investigated with Giemsa staining which showed numerous Leishmania amastigotes in all four areas. Polymerase chain reaction (PCR) test reported *Leishmania. tropica* species.

Patient received intramuscular meglumine antimoniate (glucantime) injections, 20 mg/kg/day. Improvement of both skin and oral cavity lesions was remarkable in 10 days. Because of the increase in liver enzyme, the treatment was stopped for few days and restarted with a lower dosage and continued for 28 days.

### 2.3 Outcome and follow‐up

After 28 days of treatment with glucantime, clinical improvement was significant (Figures 1 and 2). Patient was referred to rheumatologist and endocrinologist for treatment of RA and corticosteroid dependence. Subsequently, systemic corticosteroid was tapered.

## 3 DISCUSSION

Immunosuppressive treatments alter clinical manifestations of CL, and it has been shown in previous studies that immunocompromised patients experience more severe symptoms compared to the immunocompetent.^6^ Unconventional manifestations of this disease can cause a delay in diagnosis resulting in harsh circumstances such as scarring, defacement, and even disablement.^7^ The current case mimicked different conditions. The initial lesions were scaly and *erythematous* papules and plaques that covered both upper limbs, torso and distal of lower limbs which resembled psoriasis. Plaques in some areas were ulcerated with serous discharge mimicking mycobacterial infection. Some of the lesions were edematous and inflammated which alongside the poor general condition of the patient and fever resembled sweet syndrome. The oral ulcers made the primary healthcare practitioner think of herpetic infection due to immunosuppression. The association of histopathology with PCR led to diagnosis.

In this case presentation, we report a *recurrent and disseminated* CL with a very rare and unusual clinical presentation. Disseminated cutaneous leishmaniasis is a rare manifestation of CL and is linked to the cellular immunity.^4^ To the best of our knowledge, there have been three reports of disseminated CL after treatment with immunosuppressive drugs following organ transplants.^8^, ^9^, ^10^ Two cases of disseminated leishmaniasis by *Leishmania. tropica* were reported in patients with HIV in Iran which presented with multiple skin lesions on face and extremities.^11^ Also Alcover et al reported a case of diffuse CL by *Leishmania. infantum* in a patient with psoriasis and RA undergoing anti‐TNF therapy.^12^

There have been some reports on reactivation of CL infection in patients receiving immunosuppressive treatments for RA; the first case of CL reactivation was witnessed in a RA patient under treatment with systemic corticosteroids in 2005 but it was caused by *Leishmania. donovani *and presented with a single ulcerated lesion.^13^ Another report of visceral and mucocutaneous leishmaniasis recurrence was in a Belgian woman with a long history of severe RA who had been treated with etanercept, ciclosporin, and methylprednisolone.^14^ The current case correlates with earlier reports that CL can cause unconventional clinical manifestation and reactivation in patients receiving immunosuppressive treatments; however, the appearance and extent of our patient's lesions were different from any reports that have been done. Also unlike other similar studies, the patient was solely being treated with prednisolone.

The standard treatment for disseminated leishmaniasis is systemic antimonial compounds,^15^, ^16^ which in this case the aforementioned treatment course was completed and clinical improvement was witnessed.

## 4 CONCLUSION

Cutaneous leishmaniasis can cause extraordinary and unconventional clinical manifestations in immunosuppressed patients which could easily be mistaken with other diseases, and diagnosis needs further evaluations and special consideration.

## CONFLICT OF INTEREST

None declared.

## AUTHOR CONTRIBUTIONS

MM: designed and interpreted the patient data and cowrote the paper. RA: interpreted the patient data, followed up the patient, and cowrote the paper. YN and MT: designed and interpreted the patient data. VM: performed the histological examination, supervised the research, and revised the manuscript. All authors read and approved the final manuscript.

## ETHICAL APPROVAL

All procedures involving human participant were in accordance with the ethical standards of the national research committee and the 1964 Helsinki declaration and its later amendments or comparable ethical standards.

## INFORMED CONSENT

Patient signed informed consent regarding publishing their data and photographs.

## Data Availability

Data sharing is not applicable to this article as no datasets were generated or analyzed during the current study.
